# The Use of Synthetic IMU Signals in the Training of Deep Learning Models Significantly Improves the Accuracy of Joint Kinematic Predictions

**DOI:** 10.3390/s21175876

**Published:** 2021-08-31

**Authors:** Mohsen Sharifi Renani, Abigail M. Eustace, Casey A. Myers, Chadd W. Clary

**Affiliations:** Center for Orthopedic Biomechanics, University of Denver, Denver, CO 80208, USA; abigaileustace@hotmail.com (A.M.E.); Casey.Myers@du.edu (C.A.M.); Chadd.Clary@du.edu (C.W.C.)

**Keywords:** deep learning, inertial sensors, joint kinematics, synthetic data, augmentation

## Abstract

Gait analysis based on inertial sensors has become an effective method of quantifying movement mechanics, such as joint kinematics and kinetics. Machine learning techniques are used to reliably predict joint mechanics directly from streams of IMU signals for various activities. These data-driven models require comprehensive and representative training datasets to be generalizable across the movement variability seen in the population at large. Bottlenecks in model development frequently occur due to the lack of sufficient training data and the significant time and resources necessary to acquire these datasets. Reliable methods to generate synthetic biomechanical training data could streamline model development and potentially improve model performance. In this study, we developed a methodology to generate synthetic kinematics and the associated predicted IMU signals using open source musculoskeletal modeling software. These synthetic data were used to train neural networks to predict three degree-of-freedom joint rotations at the hip and knee during gait either in lieu of or along with previously measured experimental gait data. The accuracy of the models’ kinematic predictions was assessed using experimentally measured IMU signals and gait kinematics. Models trained using the synthetic data out-performed models using only the experimental data in five of the six rotational degrees of freedom at the hip and knee. On average, root mean square errors in joint angle predictions were improved by 38% at the hip (synthetic data RMSE: 2.3°, measured data RMSE: 4.5°) and 11% at the knee (synthetic data RMSE: 2.9°, measured data RMSE: 3.3°), when models trained solely on synthetic data were compared to measured data. When models were trained on both measured and synthetic data, root mean square errors were reduced by 54% at the hip (measured + synthetic data RMSE: 1.9°) and 45% at the knee (measured + synthetic data RMSE: 1.7°), compared to measured data alone. These findings enable future model development for different activities of clinical significance without the burden of generating large quantities of gait lab data for model training, streamlining model development, and ultimately improving model performance.

## 1. Introduction

Gait analysis and musculoskeletal modeling (MSM) are commonly used to quantify movement mechanics, providing insights into the diagnosis, treatment, and rehabilitation of movement disorders [1,2]. Using the current gold-standard passive-marker motion capture (MOCAP) systems, detailed kinematic measurements are time consuming, constrained to laboratory environments, and require technical expertise to generate reliable data. Wearable inertial measurement units (IMUs) enable biomechanical measurements without many of the logistical constraints of traditional techniques by translating multiple streams of IMU data into an accurate measurement of joint mechanics. However, establishing reliable clinical metrics of pathological movement with the use of IMUs remains a major hurdle.

Early IMU-based methods for measuring lower limb kinematics integrate the rotational velocity and linear acceleration data from each limb segment, coupled with orientation data from the magnetometer, to make estimations about limb segment positions and orientations [3,4]. These methods are prone to errors imparted by noise, drift, and other inaccuracies in IMU signals. More recently, the accuracy of IMU-based kinematic measurements has been improved by integration with MSMs and optimization algorithms to impose realistic joint constraints to the estimated movements [5,6,7,8,9]. These methodologies, however, require nontrivial computational resources making them less suitable for real-time applications with instantaneous feedback [6,10,11]. 

Despite the intensive computational resources necessary for training machine learning algorithms, trained models can be deployed with minimal processor power to generate instantaneous kinematic and kinetic predictions. These techniques include neural networks (NNs) to estimate ground reaction forces for gait, running and jumping [12,13,14,15,16], and lower limb joint kinematics and kinetics [10,11,15,17,18,19,20]. The accuracy of these algorithms relies on large and representative biomechanics training datasets that are frequently expensive and time consuming to collect. To expand the availability of training data, researchers are leveraging artificially generated data to improve model prediction accuracy and reliability.

The most common technique to generate artificial IMU data in movement analysis is to leverage existing passive-marker motion capture datasets to calculate simulated IMU data based on marker trajectories and accelerations [13,16,21,22,23,24]. For the purposes of this paper, artificial IMU data generated using this technique will be referred to as “simulated IMU” data. Using this technique, Mundt et al. [25] combined simulated IMU data from an archived MOCAP database with experimentally measured IMU data on a smaller subject cohort to predict lower limb kinematics and kinetics during gait. The inclusion of the simulated data in the training set reduced the root mean square error in joint kinematic estimates from 4.8° to 4.3° but did not improve joint kinetics predictions. The authors attributed the modest prediction improvements to inaccuracies in the simulated IMU data, specifically the lack of soft tissue-induced vibrations. One limitation of this technique is that observations are confined to movements measured in the lab and potentially do not span the variability present in the population at large.

Dorschky et al. [26] combined measured IMU data from subjects during walking and running with artificial IMU data generated from complementary MSMs. The authors applied perturbations to the MSM’s joint angles, ground reaction forces, and speeds based on random sampling from the experimental measures to generate synthetic IMU data for movements not observed experimentally. The constraints of the MSM and corresponding optimal control algorithm ensured the perturbations resulted in physically realistic joint mechanics. For the purposes of this paper, artificial IMU data generated for movements beyond those observed experimentally will be referred to as “synthetic IMU” data. Similar to Mundt et al. [25], the addition of the synthetic data improved kinematic predictions at the hip, knee, and ankle but had mixed effects on the joint moments and ground reaction forces. In this way, both simulated IMU data (IMU data generated from existing MoCap data) and synthetic IMU data (IMU data generated from artificial kinematics not measured in the lab) are useful for expanding model training sets, but synthetic IMU data enables expansion of the training set to uncommon movements that are difficult to measure in the lab. 

Numerical techniques to supplement existing optical tracking data with simulated IMU data or augmentation techniques to expand existing datasets with unique synthetic observations have both proven effective at enhancing kinematic predictions from machine learning algorithms. However, both techniques rely on intensive gait lab data collections, limiting widespread accessibility. In contrast, synthetic data can be generated with only a few representative gait lab measurements of an activity to establish the general kinematic patterns of the movement. It has yet to be demonstrated if machine learning models can achieve the necessary accuracy when trained exclusively with synthetic data. In this study, we aimed to develop a musculoskeletal modeling framework to augment and expand an existing dataset of gait kinematics, then use those synthetic data to train neural networks to predict 3-D joint angles from experimentally measured IMU data during gait. We hypothesize that (1) introducing synthetic IMU data into the training dataset will significantly improve the kinematics predictions and (2) models trained exclusively on synthetic data will perform equivalent to models trained using experimentally measured IMU data. 

## 2. Materials and Methods

### 2.1. IMU Measurement and Simulation Workflow Overview

**Figure 1:**
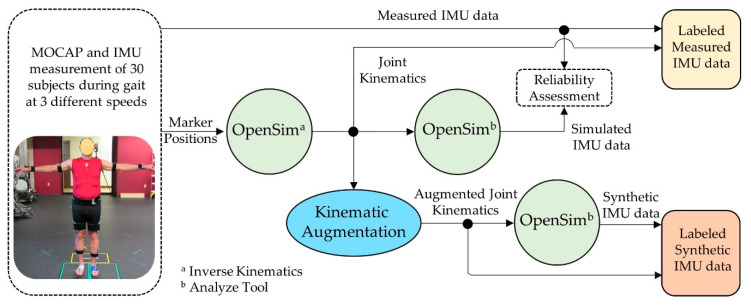
Overview of workflow for generating IMU signals labeled with joint kinematics. Knee and hip kinematic were calculated from measured marker positions using the Inverse Kinematic tool in OpenSim. The OpenSim analysis tool was used to generate simulated IMU data from the experimental kinematic and synthetic data from augmented joint kinematics. Simulated IMU signals from the experimental kinematics were compared with the measured IMU data to determine the reliability of the IMU simulation process.

In this study, we trained recurrent neural networks to predict three-dimensional hip and knee kinematics during gait using either experimentally measured IMU data, synthetically generated IMU data, or a combination of experimental and synthetic IMU data. First, combined IMU and motion capture data were collected from 30 subjects during multiple gait trials at various speeds (Figure 1). Next, the subjects’ lower limb hip and knee kinematics were calculated from the experimental marker position data, then augmented to generate synthetic IMU data via a musculoskeletal modeling workflow in OpenSim [27]. Finally, the original measured kinematic data, the synthetic kinematic data, and the combined measured and synthetic data were used to train the recurrent neural networks to estimate three-dimensional hip and knee kinematics during gait from IMU data. The detailed experimental methods can be found below.

### 2.2. Experimental Data Collection

In total, 30 subjects, including 13 subjects with OA (age = 63 ± 6, weight = 76 ± 14 kg, height = 165 ± 13 cm, 6 females and 8 males) and 17 subjects with total knee arthroplasty (age = 68 ± 5, weight = 76 ± 14 kg, height = 163 ± 13 cm, 13 females and 4 males), participated in the study as part of a larger investigation. All participants signed a consent form prior to the experiment with IRB approval (IRB# 1328728). All biomechanical measurements were carried out in the same lab setting. Subjects were outfitted with 71 reflective markers on anatomical landmarks and 17 research-grade IMUs on various limb segments and the trunk. Only IMUs located on the pelvis, left thigh, left shank, and left foot were used in this analysis [28]. Thigh and shank IMUs were attached to rigid 4-marker clusters used to track the relative orientations of the IMUs, while markers placed directly on the IMUs were used to track IMU displacements. The relative orientation of the foot IMU was tracked using markers on the medial and lateral malleoli in addition to a marker directly on the foot IMU. Similarly, the relative orientation of the pelvis IMU was tracked using markers placed on the posterior superior iliac crests and a marker on the pelvis IMU. 

**Figure 2:**
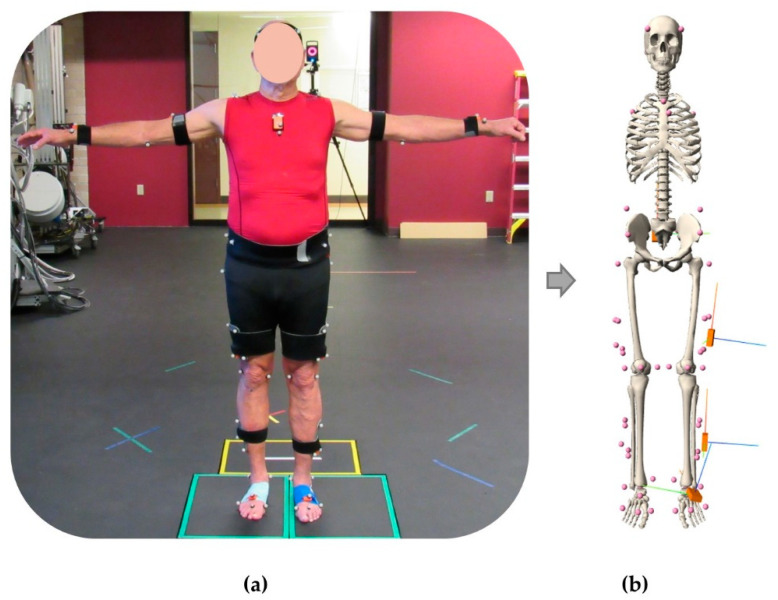
Subject fitted with reflective markers and inertial measurement units (**a**) and the corresponding musculoskeletal model representation with the virtual IMU sensors (**b**).

Subjects performed 15 trials of a 5 m walking task at three different speeds: self-selected, slow, and fast. During the walking trials, synchronized data were collected from a 13 camera Vicon motion capture system (Centennial, CO), 4 Bertec force platforms (Columbus, OH), and the IMUs (Xsens, Enschede, The Netherlands) (Figure 2a). The sampling frequency of force data, MOCAP, and IMUs (acceleration and angular velocity) were 1000 Hz, 100 Hz, and 40 Hz, respectively. The IMUs used in this study leveraged on board data processing to reduce noise and drift in the signals [29]. IMU data for each trial were upsampled using cubic interpolation to 100 Hz and filtered using a Butterworth low-pass filter with cutoff frequency of 6 Hz.

### 2.3. Musculoskeletal Modeling and IMU Simulation

Subject-specific musculoskeletal models were created for each study participant using a previously published workflow [30]. Each model included 10 rigid body segments, 23 degrees of freedom, and 92 muscle actuators. The hip and knee joints were each modeled with three rotational degrees of freedom, and the ankle was modeled as a 1 degree of freedom hinge joint. Limb segments were scaled to match the optical markers from the experiment. An inverse kinematics analysis was conducted for each subject and each gait trial using OpenSim to obtain 3-D joint kinematics at the hip and knee [27].

Four virtual IMUs were placed on the pelvis, thigh, shank, and foot according to their experimentally measured locations and orientations via fixed joints to their respective limb segments (Figure 2b). These virtual IMUs were used in subsequent steps to generate synthetic IMU signals. The angular velocities and linear accelerations of the model’s rigid segments (pelvis, thigh, and shank) from the inverse kinematics analysis were used to calculate simulated IMU signals using the Analyze Tool in OpenSim. The angular velocity of each rigid segment in the global coordinate system was transformed through the local segment’s anatomic reference frame and into the simulated IMU’s local sensor-based coordinate system to align with the experimental measurements. To calculate the simulated IMU’s acceleration, the second derivative of the position vector for the marker placed directly on the IMU was calculated in the global OpenSim coordinate system. These accelerations were transformed into the experimental IMU’s global earth-fixed coordinate system for comparison to the experimental measurements. To assess the reliability of the simulated IMU data, the root mean square error (RMSE) and the Pearson correlation coefficient (r) between the experimentally measured IMU data and the simulated IMU data were calculated.

### 2.4. Kinematic Augmentation and Synthetic IMU Data Generation

**Figure 3:**
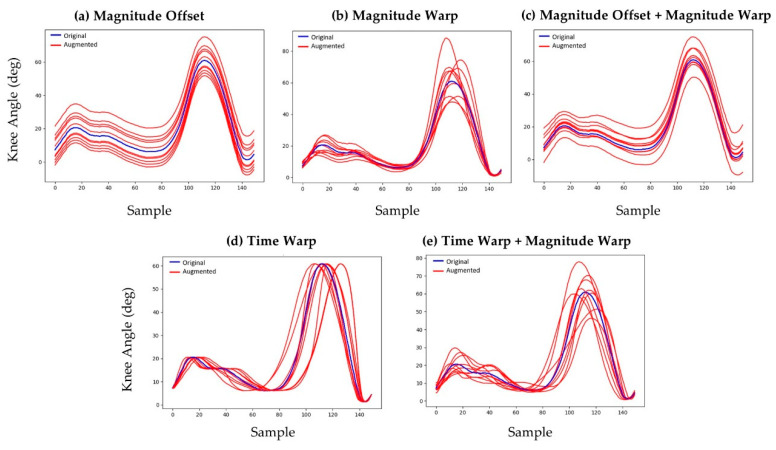
Various data augmentation methods used to generate synthetic kinematic data: (**a**) magnitude offset, (**b**) magnitude warping, (**c**) combined magnitude offset and magnitude warping, (**d**) time warping, and (**e**) combined time warping and magnitude warping.

Joint angles calculated from the measured data were segmented into individual gait cycles of the left lower limb using the heel marker resulting in 3943 unique strides from the 30 study participants. These joints angles were augmented using five different numerical techniques to introduce variation in both the magnitude of the joint angles (e.g., increased knee flexion during stance) and the timing of the gait events (e.g., shorter stance phase). These methods included magnitude offsets, magnitude warping, combinations of magnitude offsets and warping, time warping, and combinations of time warping and magnitude warping (Figure 3) [31,32,33]. 

Magnitude offsets were introduced by adding a random number from a normal distribution (µ = 0°, σ = 5°) to all joint angles from a given trial. Magnitude warping was introduced by fitting a cubic spline to seven random numbers from a normal distribution (µ = 1, σ = 0.2) that were uniformly spaced along the time domain of the input gait cycle. The cubic spline was evaluated at the time increments from the original trial’s joint angle vectors to form a distortion vector. Joint angles augmented with magnitude warping were generated by multiplying corresponding elements of the distortion vector and the original joint angle vector. The same distortion vector was used on all joint angles from a given gait trial. Augmented joint angles with combined offset and warping were generated using these same methods by first applying the magnitude warping and subsequently applying the magnitude offset.

Time warping was introduced using a similar methodology by fitting a cubic spline to seven random numbers from a normal distribution (µ = 1, σ = 0.2) that were spaced uniformly along the time domain of the input gait cycle. The cubic spline was evaluated at the time increments from the original trial’s joint angle vectors, then the cumulative sum vector was calculated and divided by the length of the original joint angle vector to form a time distortion vector. Joint angles augmented with time distortion were generated by interpolating the original joint angles at the time values in the time distortion vector. Augmented joint angles with combined time warping and magnitude warping were generated by first applying the time warp to the joint angles and then applying the magnitude warp.

One set of augmented joint kinematics was generated for every gait trial using each of the five augmentation methods described above, resulting in a total of 19,715 sets of augmented joint angles from the original 3943 measured strides (5:1 ratio). Synthetic IMU data were calculated for each set of augmented joint kinematics by using the new joint angles to animate the associated patient-specific musculoskeletal model, in lieu of optical marker locations, using the workflow described above in OpenSim Analyze. This kinematics augmentation method introduced random variation into the dataset, and no controls were implemented to ensure the resulting kinematics were strictly physiological. Kinematic perturbations were selected from normal distributions with conservative standard deviations (σ = 5° for angular offsets, 20% for magnitude warping, and 20% for time warping) that ensured the perturbations were similar to the measured kinematics. In this way, generation of the augmented kinematic data required no a priori knowledge of movement strategies, making the results more generalizable to other movements of interest and simpler to implement.

All experimentally measured IMU data and the corresponding joint angles were lowpass filtered with a second-order Butterworth filter using a cutoff frequency of 6 Hz [10,34,35]. In addition, all datasets were zero padded to a length of 200, corresponding to the maximum length of any stride in the dataset.

### 2.5. Neural Network Model Architecture, Tuning, Training, and Evaluation

To facilitate neural network model development and testing, subjects from the experimental dataset were randomly assigned into training and test groups. The training dataset included the experimental measurements for all gait cycles from 27 subjects (3451 gait cycles). All experimental measurements from the remaining three subjects were reserved for the test set (492 gait cycles).

Two independent neural network models, one for knee kinematics and one for hip kinematics, were developed to predict joint angles from the corresponding IMU data. Both networks contained a bidirectional long short-term memory (BiLSTM) layer, followed by two fully connected layers. LSTM models are a specific class of recurrent neural networks particularly suited to time series data by addressing the vanishing gradient problem [36]. Specifically, LSTM is unique in that it uses feedback to remember long-term dependencies of the input data on output data with the use of the time domain. Unlike unidirectional LSTM models, which only consider information from the past, BiLSTM models also invert the time scale of the data to consider information from the future input, which may inform the present prediction and ultimately improve accuracy [37,38]. A dropout of 0.5 was added prior to the final layer to avoid overfitting. The model input was a 200 × 24 matrix containing the three accelerations and three angular velocities from the pelvis, left thigh, left shank, and left foot IMUs. The model output was a 200 × 3 matrix containing the corresponding 3-D joint angles of the hip or knee (flexion–extension, adduction–abduction, and internal–external rotations) as a function of time. Model training was conducted using an adaptive learning rate optimization with a learning rate, beta-1, and beta-2 of 0.001, 0.9, respectively, with a total of 100 epochs [39]. The size of each batch size was 50. The model development and training were conducted using PyTorch.

The neural network models’ hyperparameters, including the number of BiLSTM layers and hidden sizes, were tuned via 5-fold cross-validation using only the experimentally measured MOCAP and IMU data in the training dataset [40]. Specifically, the training set was subdivided into 5 sets with 5–6 subjects per set. In each fold, one set was reserved for validation, while the remaining four were used to train models with all combinations of hyperparameters. The prediction accuracies of these models and corresponding hyperparameters were evaluated on the validation set designated for that fold. Hyperparameters that resulted in the minimum average RMSE across all five folds for each model were used in all subsequent model training and evaluation (Table 1). The optimal hip model had 1 layer with a hidden size of 32, while the optimal knee model had 1 layer with a hidden size of 128.

To investigate the influence of synthetic IMU and lower limb kinematic data on prediction accuracy, hip and knee neural network models were trained on three variations of the training dataset. The first variation included the experimentally measured IMU signals and associated kinematics for gait cycles from all 27 subjects included in the training cohort (3451 measured gait cycles). The second variation included only the synthetic IMU signals and associated kinematics generated from the subjects in the training cohort (17,255 synthetic gait cycles). The third dataset included the measured data from the training cohort and the corresponding synthetic data generated for those same subjects (3451 measured gait cycles and 17,255 synthetic gait cycles). All three sets of trained models were used to predict the lower limb joint angles for all trials of the three subjects assigned to the test cohort using the measured IMU data (492 measured gait cycles). The predictive accuracy of the models was quantified by calculating the RMSE, normalized-RMSE, and Pearson correlation coefficients between the predicted and measured joint angles from the test set. A multivariate analysis of variance (MANOVA) was performed with the predicted RMSE for each of the six kinematic degrees of freedom (e.g., Knee Flex–Ext or Hip Ad–Ab) as the dependent variables and the training dataset type as the independent variable. Tukey’s honest significant different (HSD) post hoc tests were performed to determine which kinematic degrees of freedom demonstrated statistically significant prediction improvements with each training dataset (*p* < 0.05).

## 3. Results

### 3.1. Simulated IMU Accuracy

**Figure 4:**
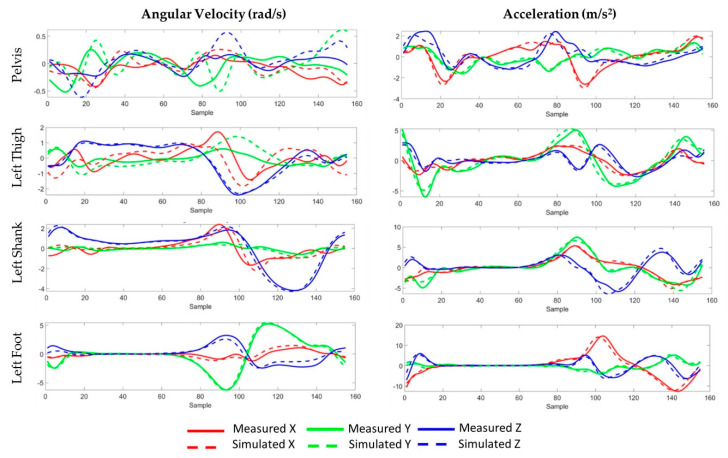
Measured (solid) and simulated (dashed) 3-D angular velocity and acceleration data during an exemplary gait cycle. The IMU sensors were located on the pelvis, the lateral left thigh, the lateral left shank, and on top of the left foot.

The average RMSE between the measured IMU data and simulated IMU based on marker trajectories across all sensors for angular velocities was 0.56 rad/s (ranging from 0.33 to 1.02) with correlation coefficients ranging from 0.29 in the pelvis sensor’s *y*-axis to 0.98 in the foot sensor’s *y*-axis (Table 2, Figure 4). Similarly, the average RMSE for accelerations was 1.43 m/s^2^ (ranging from 0.62 to 2.46) with correlation coefficients ranging from 0.75 in the thigh sensor’s *y*-axis to 0.96 in the shank sensor’s *x*-axis. IMU predictions for free accelerations were generally more accurate than angular velocities with average correlations coefficients of 0.86, compared to 0.71, respectively. Predictions for the pelvis IMU were consistently worse than predictions for the other segments, particularly for the pelvis rotational velocities (mean r = 0.47).

### 3.2. Model Accuracy

**Figure 5:**
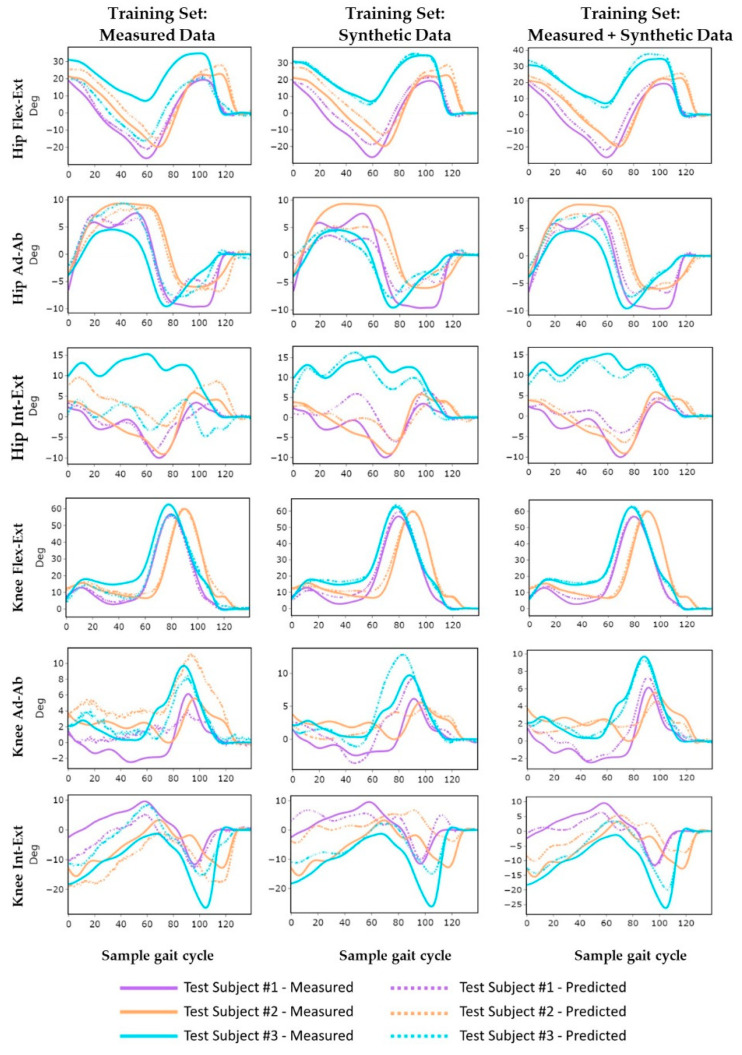
Measured and predicted lower limb kinematics in each degree of freedom during an exemplary trial for each test subject during gait: prediction (dashed line) and measured (solid line) for different training datasets.

Inclusion of synthetic kinematics to supplement the measured data in the neural network training dataset statistically significantly improved kinematic predictions for all hip and knee degrees of freedom (*p* < 0.001, Table 3, Figure 5). Likewise, the neural networks trained exclusively on synthetic data significantly improved prediction accuracy compared to models trained exclusively on measured data for five of the six kinematic degrees of freedom, excluding knee adduction–abduction (Ad–Ab) (*p* < 0.002). The mean RMSE and correlation coefficients for hip kinematics improved from 4.5° ± 1.6° and 0.82 ± 0.13 when trained on measured data to 2.3° ± 0.3° and 0.91 ± 0.08 when trained on synthetic data, corresponding to a 38% reduction in RMSE and a 13% increase in the correlation coefficient. Predictions improved to 1.9° ± 0.2° and 0.96 ± 0.03 when trained on both measured and synthetic data together, corresponding to a 54% reduction in RMSE and a 20% improvement in correlation coefficient, compared to the measured data alone. Mean RMSE and correlation coefficients for knee kinematic predictions followed a similar trend, improving from 3.3 ± 0.2° and 0.83 ± 0.12 when trained on experimental data to 2.9 ± 0.7° and 0.84 ± 0.12 for synthetic data, and 1.7 ± 0.4° and 0.96 ± 0.04 for the combined training dataset. 

Across all joint angle predictions, the models consistently had the highest accuracy when predicting knee flexion–extension (Flex–Ext) with correlation coefficients of greater than 0.99 ± 0.01 and nRMSE ranging from 1.9 ± 0.7 to 3.9 ± 1.6 across all training datasets. Conversely, the hip and knee internal–external (Int–Ext) rotation predictions consistently had the lowest accuracy, with nRMSE ranging from 9.8 ± 3.5 to 23.9 ± 11.1 for the hip, and from 14.1 ± 6.4 to 25.5 ± 6.3 for the knee. The models trained on both measured and synthetic data had the highest generalizability with the lowest standard deviations in prediction errors for patients in the test cohort (maximum standard deviations were 0.07 for r, 1.6° for RMSE, and 6.8 for nRMSE). Accuracy metrics for individual subjects in the test cohort are reported in Appendix A.

## 4. Discussion

This study demonstrated a musculoskeletal modeling-based workflow to generate synthetic kinematic data that were used to improve the performance of neural networks to predict 3-D hip and knee rotations during gait. Supplementation of the measured kinematic training data with synthetic data that had been augmented in both magnitude and timing reduced the prediction RMSE by 54% at the hip and 45% at the knee. Training the model with synthetic data resulted in prediction accuracy that was either equivalent to or better than training purely on experimentally measured data for all three kinematic degrees of freedom at both the hip and knee. 

Synthetic data used in model development must preserve the physical relationship between joint rotations and the corresponding IMU data. The musculoskeletal workflow used in the current study to generate simulated IMU data had mixed results across different limb segments. We saw the worst correlations between measurements and simulations in the pelvis rotational velocities. The magnitudes of the pelvis rotational velocities were also considerably smaller than the other limb segments during gait. Inclusion of activities that require greater pelvic rotational velocities into the training set may improve overall predictive accuracy. Additionally, rigidly attaching the pelvis IMU was challenging given the amount of soft tissue present on some study participants. These factors likely caused soft tissue artifacts to have a larger effect on the pelvis IMU measurements than the other limb segments. Angular velocity correlation coefficients for the other limb segments averaged greater than 0.79 across the three degrees of freedom. Despite the limitation associated with synthetic pelvic rotational velocities, the inclusion of the synthetic data considerably improved the predictive ability of the model, even for the hip joint angles. This result suggests that some components of the IMU signals contribute less to the overall prediction accuracy. Future work to determine the most basic set of IMU data necessary to accurately predict joint rotations would be valuable for guiding hardware development for commercial systems used in performance monitoring or rehabilitation. 

Data augmentation is commonly used to expand training datasets for machine learning algorithms. Most augmentation approaches for IMU-based applications employ label-preserving transformations such as adding noise or simulating variation in sensor positioning [31]. More recently, Dorschky et al. published a non-label preserving augmentation method in which planer musculoskeletal models with an optimal control simulation framework generated synthetic IMU data coupled with 2-D joint kinematics and kinetics [26]. The optimal control simulation was necessary to preserve the physical relationship between joint kinematics, ground reaction forces, and the calculated joint kinetics but required computationally expensive and specialized modeling techniques, which may not be necessary for improving kinematic prediction accuracy. In the current study, we randomly augmented the joint kinematics with variations in both time and magnitude but did not implement controls to ensure the resulting joint kinematics were physically realistic. Additionally, the augmentation was implemented using a freely available musculoskeletal modeling framework. Therefore, these methods may be easier to implement for non-specialists in biomechanics and provide substantial time savings in the generation of valuable synthetic data for other applications. While the inclusion of unphysiological synthetic data in the training set still enabled significant improvements of prediction accuracy, it is unclear if this would hold true of other types of movements. Future work should consider methods to generate more targeted and realistic synthetic data that span the variability in the subject population of interest such as the meta-learning algorithm proposed by Ruiz et al. [6,41].

Differences in subject characteristics, activities, dataset size, sensor type and configuration, and variation in reported data make direct comparisons of prediction accuracy with previous studies tenuous. Mundt et al. reported 3-D hip and knee joint angle predictions from a large cohort of over 88,000 cycles of simulated IMU data validated with more recent experimental IMU measurements and achieved comparable results to the current study (Table 4 and Table 5) with approximate average hip and knee RMSEs of 1.9° and 1.7°, respectively [25]. Similar to the current study, they also observed higher prediction correlations for hip and knee flexion–extension, compared to rotations in the frontal and axial planes. While both studies used an LSTM model architecture, the current study required significantly fewer experimental observations to achieve an equivalent level of prediction accuracy. Rapp et al. predicted hip and knee joint angles during gait in 420 subjects using an LSTM model coupled with an optimization algorithm to account for differences in the predicted and measured segment rotational velocities [42]. When evaluated on simulated IMU data, the combined algorithm achieved an RMSE of 4.2° at the knee and 4.1° at the hip prior to a calibration step, which further improved the accuracy. Dorschky et al. reported RMSEs in hip and knee flexion of 5.1° and 4.8°, respectively, which were higher than the current study, but included predictions for both gait and running at different speeds using a smaller training set of only 418 measured cycles and individual model for each joint angle prediction [26]. Gholami et al. used a single IMU mounted to the foot of 10 subjects to predict hip and knee flexion during treadmill running with RMSEs of 5.6° and 6.5°, respectively [10]. It remains unclear whether the higher accuracy achieved in the current study was due to the larger experimental dataset or that higher accelerations and rotational velocities during running make predictions more difficult. 

We considered kinematics from the optical motion capture system as the ground truth for training and subsequent accuracy assessment; however, uncertainty in marker placement, skin artifacts, and measurement errors limit the achievable accuracy of these systems. Benoit et al. reported absolute errors in knee kinematics due to soft tissue artifacts between 2.4° and 2.8° for knee flexion, 2.5 and 4.4° for knee adduction–abduction, and 2.2° and 2.8° for knee internal–external rotations during gait [44]. In a previous study, we quantified the 5–95 percent uncertainty bounds in kinematic measurements based on input uncertainty in marker locations and movement artifacts using the current musculoskeletal modeling workflow [45]. Knee flexion had the smallest uncertainty bounds (2.7 ± 0.3°), while uncertainty in hip adduction–abduction (3.0 ± 0.3°), internal–external (5.1 ± 1.0°), and flexion–extension (6.4 ± 0.5) rotations were higher. In contrast, the RMSE in prediction accuracy achieved using the combined training set of the current study ranged from 33% to 52% of the reported uncertainty bounds for corresponding joint angles. Given this level of uncertainty, the performance of the current neural networks is well within the uncertainty in the measurement techniques. Future improvements in model performance will require higher accuracy training data.

Selection of the appropriate neural network architecture is an important step in attaining the requisite accuracy for model predictions. In previous work, we systematically evaluated multiple neural network configurations for predicting spatiotemporal gait characteristics on this same dataset and found that convolutional neural networks yielded the highest accuracy predictions [28]. Mundt et al. also compared LSTM and feedforward neural networks (FFNN) performance on time-normalized gait cycle input data and achieved better performance using the FFNN. In a more recent study, the same group compared the performance of three common neural networks for joint kinematic and kinetic predictions, finding that convolutional neural networks achieved higher accuracy than LSTM networks but required additional data processing steps that would hinder applications working in real time [46]. For the current study, we evaluated multiple network architectures [10,22,26,47] and found the BiLSTM model had the most robust performance. Unlike convolutional neural networks, which require input data of consistent length, recurrent neural networks such as LSTM are time independent and accept input data of arbitrary length. This approach reduced the necessary preprocessing of data (e.g., normalizing cycle to % gait) and the associated time required to build the neural networks [26,43]. We used zero padding to accommodate the time dependency of the gait cycles, which led to better performance on LSTM-based networks [20,22,36].

The finding that equivalent predictive accuracy can be achieved when training neural networks using synthetic kinematic data, in contrast to experimental data, expands the speed and accessibility of model development. Previously, the generation of experimental training data was the bottleneck for algorithm development and required significant investments of time and capital equipment. The current results demonstrate that reasonable predictive accuracy can be achieved using a cohort of musculoskeletal models, representative joint angles for the activities of interest, and a robust pipeline for generating simulated IMU data. The generalizability of this workflow to additional movements, particularly more dynamic movements with higher variability (i.e., stair descent, running, or cutting maneuvers), is still unclear and requires further validation. 

This study had a few notable limitations that should be considered when evaluating the results. All the subjects who participated in this study had either end-stage osteoarthritis in the hip or knee or had recently recovered from a total joint arthroplasty. This patient population has been shown to exhibit gait adaptations, including a slower pace, shorter step length, reduced knee flexion, and increased levels of variability that may affect the generalization of the model to healthy individuals [28,48,49,50]. Gait measurements were taken in the laboratory environment, which may affect the subjects’ normal gait patterns. Research grade IMUs were used in this study that had on board data processing to reduce noise and drift in the signals [29]. These IMUs were placed in specific, repeatable anatomic positions to minimize variability associated with sensor positioning. Additional simulation and model training would be necessary to make the system robust to noise from lower-grade sensors and increased variability in sensor positioning on the limb segments so the system could be deployable in real-life unsupervised applications. Finally, model hyperparameter selection was based solely on the measured data and not evaluated using synthetic data. While we anticipate that incorporating synthetic data into the hyperparameter selection would further improve model accuracy when trained with synthetic data, this has yet to be demonstrated. 

## 5. Conclusions

The present study demonstrated that recurrent neural network predictions of 3-D hip and knee angles during gait using IMU sensors can be significantly improved using synthetic kinematic and IMU data. On average, RMSEs in joint angle predictions were improved by 38% at the hip and 11% at the knee when models were trained on synthetic data, compared to measured data alone. When models were trained on both measured and synthetic data, RMSEs were reduced by 54% at the hip and 45% at the knee, compared to measured data alone. The musculoskeletal workflow described here enables future model development for other activities that have clinical significance without the burden of generating large quantities of gait lab data for model training.

## Figures and Tables

**Table 1:** Architecture of bidirectional long short-term memory neural networks with tuned hyperparameters.

Model	*n*-Layers Evaluated	Hidden Sizes Evaluated	Optimal *n*-Layer	Optimal Hidden Size
Hip-BiLSTM	1, 2, 3, 4	16, 32, 64, 96, 128	1	32
Knee-BiLSTM	1, 2, 3, 4	16, 32, 64, 96, 128	1	128

**Table 2:** Pearson correlation coeffecient (r) and root mean square error (RMSE) and of angular velocity and acceleration between simulated and measured IMU data across all subjects and trials.

Segment	IMU DoF	Angular Velocity (rad/s)			Acceleration (m/s^2^)		
		r	RMSE	nRMSE	r	RMSE	nRMSE
		(Mean ± Std)	(Mean ± Std)	(Mean ± Std)	(Mean ± Std)	(Mean ± Std)	(Mean ± Std)
Pelvis	x	0.62 ± 0.15	0.40 ± 0.17	19.47 ± 4.90	0.88 ± 0.10	0.65 ± 0.30	11.03 ± 3.79
	y	0.29 ± 0.24	0.36 ± 0.15	32.28 ± 11.00	0.79 ± 0.12	0.62 ± 0.23	12.15 ± 3.02
	z	0.52 ± 0.32	0.46 ± 0.21	26.58 ± 9.02	0.86 ± 0.11	0.75 ± 0.33	11.16 ± 3.76
Left Thigh	x	0.67 ± 0.13	0.71 ± 0.19	16.22 ± 4.39	0.88 ± 0.10	1.64 ± 0.71	9.19 ± 3.37
	y	0.61 ± 0.23	0.48 ± 0.16	23.64 ± 8.01	0.75 ± 0.23	1.12 ± 0.59	11.45 ± 4.90
	z	0.95 ± 0.05	0.40 ± 0.20	8.56 ± 4.03	0.84 ± 0.12	1.17 ± 0.59	9.79 ± 3.23
Left Shank	x	0.83 ± 0.11	0.63 ± 0.17	10.5 ± 3.67	0.96 ± 0.03	1.51 ± 0.70	5.32 ± 1.90
	y	0.85 ± 0.21	0.33 ± 0.15	11.79 ± 7.33	0.81 ± 0.19	1.51 ± 0.82	9.82 ± 4.95
	z	0.98 ± 0.02	0.47 ± 0.18	5.22 ± 1.81	0.92 ± 0.05	1.31 ± 0.51	8.00 ± 2.12
Left Foot	x	0.39 ± 0.41	1.02 ± 0.34	20.24 ± 8.14	0.95 ± 0.04	2.46 ± 1.02	5.66 ± 2.11
	y	0.98 ± 0.02	0.69 ± 0.27	4.69 ± 1.53	0.85 ± 0.15	2.15 ± 1.15	8.39 ± 4.08
	z	0.85 ± 0.17	0.79 ± 0.24	11.16 ± 4.49	0.87 ± 0.08	2.33 ± 1.10	9.30 ± 2.65

**Table 3:** Model prediction accuracy for hip and knee joint angles with different sets of training data: measured data, synthetic data, and combined measured and synthetic data. Accuracy metrics include the Pearson correlation coefficient (r), root mean square error (RMSE), and normalized root mean square error (nRMSE).

**Training Set**	**# Samples**	**Hip Flex–Ext**			**Hip Ad–Ab**			**Hip Int–Ext**			**Hip Average**		
		**r**	**RMSE (°)**	**nRMSE**	**r**	**RMSE (°)**	**nRMSE**	**r**	**RMSE (°)**	**nRMSE**	**r**	**RMSE (°)**	**nRMSE**
Measured	3943	0.88 ± 0.12	7.2 ± 5.0	15.4 ± 10.8	0.94 ± 0.04	2.1 ± 0.7	10.1 ± 3.2	0.64 ± 0.24	4.2 ± 2.0	23.9 ± 11.1	0.82 ± 0.13	4.5 ± 1.6	16.5 ± 8.4
Synthetic	17,255	0.98 ± 0.03	2.6 ± 1.5	5.7 ± 3.2	0.95 ± 0.05	2.0 ± 0.6	9.5 ± 2.9	0.81 ± 0.17	2.3 ± 0.8	12.8 ± 4.6	0.91 ± 0.08	2.3 ± 0.3	9.3 ± 3.6
Measured + Synthetic	20,706	0.98 ± 0.01	2.6 ± 1.1	5.5 ± 2.3	0.98 ± 0.02	1.3 ± 0.5	6.1 ± 2.2	0.93 ± 0.07	1.7 ± 0.6	9.8 ± 3.5	0.96 ± 0.03	1.9 ± 0.2	7.1 ± 2.7
**Training Set**	**# Samples**	**Knee Flex–Ext**			**Knee Ad–Ab**			**Knee Int–Ext**			**Knee Average**		
		**r**	**RMSE (°)**	**nRMSE**	**r**	**RMSE (°)**	**nRMSE**	**r**	**RMSE (°)**	**nRMSE**	**r**	**RMSE (°)**	**nRMSE**
Measured	3943	0.99 ± 0.01	2.9 ± 1.1	3.9 ± 1.6	0.75 ± 0.22	2.0 ± 0.8	15.2 ± 6.3	0.77 ± 0.14	7.0 ± 1.8	25.5 ± 6.3	0.83 ± 0.12	3.3 ± 0.2	14.9 ± 4.7
Synthetic	17,255	0.99 ± 0.01	2.1 ± 0.6	2.9 ± 0.8	0.82 ± 0.13	2.0 ± 0.6	15.1 ± 4.5	0.70 ± 0.24	6.4 ± 2.8	24.0 ± 11.3	0.84 ± 0.12	2.9 ± 0.7	14.0 ± 5.5
Measured + Synthetic	20,706	0.99 ± 0.01	1.4 ± 0.5	1.9 ± 0.7	0.94 ± 0.06	1.2 ± 0.4	6.6 ± 2.3	0.93 ± 0.07	3.8 ± 1.6	14.1 ± 6.4	0.96 ± 0.04	1.7 ± 0.4	7.5 ± 3.1

**Table 4:** Reported prediction accuracy compared with previous studies for hip kinematics. Sensor configurations included pelvis (P), thigh (T), shank (S), and foot (F). Synthetic data were data generated by augmented kinematics data, while simulated data were generated by existing motion capture data.

DoF	Reference	Sensor Configuration	# Subjects	# Cycles	Data Type	Activity	r	RMSE(°)	nRMSE
**Hip Flex–Ext**	Current	P T S F	27	3943 + 17,255	Measured + Synthetic	Gait	0.98	2.6	5.5
	Mundt 2020a (PS-Net) [43]	P S	115	88,067	Simulated	Gait	0.98	1.6	NR
	Mundt 2020b (FFNN) [25]	P T S	93	3098 + 46,437	Measured + Simulated	Gait	0.99	5.2	NR
	Mundt 2019 (FFNN) [22]	P T S F	75	1028	Simulated	Gait	0.99	1.3	NR
	Dorschky 2020 (CNN) [26]	P T S F	7	418 + 6688	Measured + Synthetic	Gait and Running	1	5.1	NR
	Rapp 2021 (LSTM) [42]	P T S F	420	NR	Simulated	Gait	NR	4.3	NR
	Gholami 2020 (CNN) [10]	F	10	NR	Simulated	Running	0.8	5.6	9.9
**Hip Ad–Ab**	Current	P T S F	27	3943 + 17,255	Measured + Synthetic	Gait	0.98	1.3	6.1
	Mundt 2020a (PS-Net)	P S	115	88,067	Simulated	Gait	0.94	0.9	NR
	Mundt 2020b (FFNN)	P T S	93	3098 + 46,437	Measured + Simulated	Gait	0.96	2.1	NR
	Mundt 2019 (FFNN)	P T S F	75	1028	Simulated	Gait	0.98	1.3	NR
	Rapp 2021 (LSTM)	P T S F	420	NR	Simulated	Gait	NR	2.7	NR
**Hip Int–Ext**	Current	P T S F	27	3943 + 17,255	Measured + Synthetic	Gait	0.93	1.7	9.8
	Mundt 2020a (PS-Net)	P S	115	88,067	Simulated	Gait	0.64	2.1	NR
	Mundt 2020b (FFNN)	P T S	93	3098 + 46,437	Measured + Simulated	Gait	0.88	5.2	NR
	Mundt 2019 (FFNN)	P T S F	75	1028	Simulated	Gait	0.86	2.5	NR
	Rapp 2021 (LSTM)	P T S F	420	NR	Simulated	Gait	NR	5.2	NR
**Hip Average**	Current	P T S F	27	3943 + 17,255	Measured + Synthetic	Gait	0.96	1.9	7.1
	Mundt 2020a (PS-Net)	P S	115	88,067	Simulated	Gait	0.85	1.5	NR
	Mundt 2020b (FFNN)	P T S	93	3098 + 46,437	Measured + Simulated	Gait	0.94	4.2	NR
	Mundt 2019 (FFNN)	P T S F	75	1028	Simulated	Gait	0.94	1.7	NR
	Rapp 2021 (LSTM)	P T S F	420	NR	Simulated	Gait	NR	4.1	NR

**Table 5:** Reported prediction accuracy compared with previous studies for knee kinematics. Sensor configurations included pelvis (P), thigh (T), shank (S), and foot (F). Synthetic data were data generated by augmented kinematics data, while simulated data were generated by existing motion capture data.

DoF	Reference	Sensor Configuration	# Subjects	# Cycles	Data Type	Activity	r	RMSE(°)	nRMSE
**Knee Flex–Ext**	Current	P T S F	27	3943 + 17,255	Measured + Synthetic	Gait	0.99	1.4	1.9
	Mundt 2020a (PS-Net)	P S	115	88,067	Simulated	Gait	0.99	1.7	NR
	Mundt 2020b (FFNN)	P T S	93	3098 + 46,437	Measured + Simulated	Gait	0.98	4.5	NR
	Mundt 2019 (FFNN)	P T S F	75	1028	Simulated	Gait	0.99	1.4	NR
	Dorschky 2020 (CNN)	P T S F	7	418 + 6688	Measured + Synthetic	Gait & Running	0.99	4.8	NR
	Rapp 2021 (LSTM)	P T S F	420	NR	Simulated	Gait	NR	3.1	NR
	Gholami 2020 (CNN)	F	10	NR	Simulated	Running	0.93	6.5	6.5
**Knee Ad–Ab**	Current	P T S F	27	3943 + 17,255	Measured + Synthetic	Gait	0.94	1.2	6.6
	Mundt 2020a (PS-Net)	P S	115	88,067	Simulated	Gait	0.95	1.5	NR
	Mundt 2020b (FFNN)	P T S	93	3098 + 46,437	Measured + Simulated	Gait	0.80	2.5	NR
	Mundt 2019 (FFNN)	P T S F	75	1028	Simulated	Gait	0.79	1.6	NR
	Rapp 2021 (LSTM)	P T S F	420	NR	Simulated	Gait	NR	3.2	NR
**Knee Int–Ext**	Current	P T S F	27	3943 + 17,255	Measured + Synthetic	Gait	0.93	2.8	14.1
	Mundt 2020a (PS-Net)	P S	115	88,067	Simulated	Gait	0.93	2.5	NR
	Mundt 2020b (FFNN)	P T S	93	3098 + 46,437	Measured + Simulated	Gait	0.97	5.5	NR
	Mundt 2019 (FFNN)	P T S F	75	1028	Simulated	Gait	0.95	1.7	NR
	Rapp 2021 (LSTM)	P T S F	420	NR	Simulated	Gait	NA	6.4	NR
**Knee Average**	Current	P T S F	27	3943 + 17,255	Measured + Synthetic	Gait	0.96	1.7	7.5
	Mundt 2020a (PS-Net)	P S	115	88,067	Simulated	Gait	0.95	1.9	NR
	Mundt 2020b (FFNN)	P T S	93	3098 + 46,437	Measured + Simulated	Gait	0.92	4.2	NR
	Mundt 2019 (FFNN)	P T S F	75	1028	Simulated	Gait	0.91	1.6	NR
	Rapp 2021 (LSTM)	P T S F	420	NR	Simulated	Gait	NA	4.2	NR

## Appendix A. Prediction Accuracy for Each of the Three Subjects in the Test Cohort

**Test Subject #1**													
**Training Set**	**# Samples**	**Hip Flex–Ext**			**Hip Ad–Ab**			**Hip Int–Ext**			**Hip Average**		
		**r**	**RMSE (°)**	**nRMSE**	**r**	**RMSE (°)**	**nRMSE**	**r**	**RMSE (°)**	**nRMSE**	**r**	**RMSE (°)**	**nRMSE**
Measured	3943	0.97 ± 0.02	3.4 ± 1.1	7.3 ± 2.4	0.94 ± 0.04	2.0 ± 0.6	9.4 ± 3.0	0.84 ± 0.08	2.0 ± 0.6	11.2 ± 3.4	0.92 ± 0.04	2.4 ± 0.8	9.3 ± 2.9
Synthetic	17,255	0.97 ± 0.04	0.97 ± 0.04	3.7 ± 1.7	0.94 ± 0.09	2.2 ± 0.7	10.6 ± 3.3	0.70 ± 0.15	2.7 ± 0.8	15.3 ± 4.6	0.87 ± 0.09	2.9 ± 1.1	11.2 ± 3.8
Measured + Synthetic	20,706	0.97 ± 0.01	0.97 ± 0.01	3.5 ± 0.9	0.96 ± 0.02	1.6 ± 0.4	7.4 ± 1.8	0.87 ± 0.08	2.0 ± 0.5	11.1 ± 2.8	0.94 ± 0.04	2.4 ± 0.6	8.7 ± 2.2
**Training Set**	**# Samples**	**Knee Flex–Ext**			**Knee Ad–Ab**			**Knee Int–Ext**			**Knee Average**		
		**r**	**RMSE (°)**	**nRMSE**	**r**	**RMSE (°)**	**nRMSE**	**r**	**RMSE (°)**	**nRMSE**	**r**	**RMSE (°)**	**nRMSE**
Measured	3943	0.99 ± 0.01	2.2 ± 0.7	3.0 ± 0.9	0.49 ± 0.20	1.9 ± 0.5	14.3 ± 3.6	0.69 ± 0.11	4.6 ± 0.9	23.5 ± 4.6	0.72 ± 0.10	2.9 ± 0.7	13.6 ± 30.0
Synthetic	17,255	0.99 ± 0.01	2.3 ± 0.7	3.1 ± 0.9	0.76 ± 0.10	2.2 ± 0.6	16.9 ± 4.3	0.85 ± 0.06	2.6 ± 0.4	13.3 ± 2.3	0.87 ± 0.06	2.4 ± 0.6	11.1 ± 2.5
Measured + Synthetic	20,706	0.99 ± 0.01	1.6 ± 0.4	2.2 ± 0.6	0.91 ± 0.06	0.9 ± 0.3	7.0 ± 2.0	0.94 ± 0.04	2.1 ± 0.5	10.5 ± 2.7	0.95 ± 0.03	1.5 ± 0.4	6.6 ± 1.8

**Test Subject #2**													
**Training Set**	**# Samples**	**Hip Flex–Ext**			**Hip Ad–Ab**			**Hip Int–Ext**			**Hip Average**		
		**r**	**RMSE (°)**	**nRMSE**	**r**	**RMSE (°)**	**nRMSE**	**r**	**RMSE (°)**	**nRMSE**	**r**	**RMSE (°)**	**nRMSE**
Measured	3943	0.96 ± 0.05	3.6 ± 1.5	7.6 ± 3.1	0.96 ± 0.04	1.9 ± 0.6	9.1 ± 2.9	0.49 ± 0.20	5.0 ± 1.2	28.1 ± 6.6	0.80 ± 0.10	3.5 ± 1.1	15.0 ± 4.2
Synthetic	17,255	0.98 ± 0.02	2.9 ± 1.2	6.3 ± 2.6	0.96 ± 0.03	2.3 ± 0.4	10.8 ± 2.0	0.74 ± 0.15	2.7 ± 0.5	15.3 ± 2.9	0.89 ± 0.06	2.6 ± 0.7	10.8 ± 2.5
Measured + Synthetic	20,706	0.99 ± 0.01	1.9 ± 0.7	4.0 ± 1.6	0.99 ± 0.01	1.2 ± 0.4	5.7 ± 1.9	0.91 ± 0.07	2.0 ± 0.6	11.2 ± 3.3	0.96 ± 0.03	1.7 ± 0.6	7.0 ± 2.3
**Training Set**	**# Samples**	**Knee Flex–Ext**			**Knee Ad–Ab**			**Knee Int–Ext**			**Knee Average**		
		**r**	**RMSE (°)**	**nRMSE**	**r**	**RMSE (°)**	**nRMSE**	**r**	**RMSE (°)**	**nRMSE**	**r**	**RMSE (°)**	**nRMSE**
Measured	3943	0.99 ± 0.01	2.7 ± 1.4	3.6 ± 1.8	0.80 ± 0.09	2.9 ± 0.7	22.1 ± 5.1	0.89 ± 0.07	4.4 ± 1.3	22.0 ± 6.7	0.89 ± 0.06	3.3 ± 1.1	15.9 ± 4.5
Synthetic	17,255	0.99 ± 0.01	2.0 ± 0.5	2.7 ± 0.6	0.73 ± 0.10	1.5 ± 0.4	11.5 ± 2.7	0.81 ± 0.13	3.8 ± 1.1	19.3 ± 5.7	0.84 ± 0.08	2.4 ± 0.6	11.1 ± 3.0
Measured + Synthetic	20,706	0.99 ± 0.01	1.2 ± 0.5	1.6 ± 0.7	0.91 ± 0.06	0.8 ± 0.3	6.2 ± 2.2	0.96 ± 0.03	2.1 ± 0.6	10.5 ± 3.3	0.96 ± 0.03	1.4 ± 0.5	6.1 ± 2.0

**Test Subject #3**													
**Training Set**	**# Samples**	**Hip Flex–Ext**			**Hip Ad–Ab**			**Hip Int–Ext**			**Hip Average**		
		**r**	**RMSE (°)**	**nRMSE**	**r**	**RMSE (°)**	**nRMSE**	**r**	**RMSE (°)**	**nRMSE**	**r**	**RMSE (°)**	**nRMSE**
Measured	3943	0.74 ± 0.08	13.3 ± 1.9	28.6 ± 4.1	0.93 ± 0.03	2.4 ± 0.6	11.5 ± 3.1	0.62 ± 0.25	5.4 ± 1.7	30.7 ± 9.4	0.76 ± 0.12	7.0 ± 1.4	23.6 ± 5.5
Synthetic	17,255	0.99 ± 0.01	1.6 ± 0.5	3.5 ± 1.0	0.95 ± 0.03	1.6 ± 0.4	7.5 ± 1.9	0.96 ± 0.02	1.5 ± 0.4	8.7 ± 2.3	0.97 ± 0.02	1.6 ± 0.4	6.5 ± 1.8
Measured + Synthetic	20,706	0.99 ± 0.01	2.4 ± 0.8	5.1 ± 1.7	0.98 ± 0.01	1.1 ± 0.5	5.3 ± 2.2	0.98 ± 0.01	1.3 ± 0.5	7.6 ± 3.0	0.98 ± 0.01	1.6 ± 0.6	6.0 ± 2.3
**Training Set**	**# Samples**	**Knee Flex–Ext**			**Knee Ad–Ab**			**Knee Int–Ext**			**Knee Average**		
		**r**	**RMSE (°)**	**nRMSE**	**r**	**RMSE (°)**	**nRMSE**	**r**	**RMSE (°)**	**nRMSE**	**r**	**RMSE (°)**	**nRMSE**
Measured	3943	0.98 ± 0.01	3.6 ± 0.8	4.9 ± 1.0	0.91 ± 0.03	1.3 ± 0.2	10.1 ± 1.8	0.72 ± 0.12	5.9 ± 0.8	30.1 ± 3.9	0.87 ± 0.05	3.6 ± 0.6	15.0 ± 2.3
Synthetic	17,255	0.99 ± 0.01	2.1 ± 0.6	2.8 ± 0.8	0.95 ± 0.02	2.2 ± 0.5	16.9 ± 4.2	0.49 ± 0.24	7.3 ± 1.1	36.7 ± 5.8	0.81 ± 0.09	3.8 ± 0.8	18.8 ± 3.6
Measured + Synthetic	20,706	0.99 ± 0.01	1.4 ± 0.4	1.8 ± 0.6	0.98 ± 0.01	0.9 ± 0.3	6.5 ± 2.6	0.90 ± 0.09	4.0 ± 1.2	20.2 ± 5.9	0.96 ± 0.04	2.1 ± 0.7	9.5 ± 3.0
